# Brown Algae Carbohydrates: Structures, Pharmaceutical Properties, and Research Challenges

**DOI:** 10.3390/md19110620

**Published:** 2021-10-31

**Authors:** Yanping Li, Yuting Zheng, Ye Zhang, Yuanyuan Yang, Peiyao Wang, Balázs Imre, Ann C. Y. Wong, Yves S. Y. Hsieh, Damao Wang

**Affiliations:** 1 College of Food Science, Southwest University, Chongqing 400715, China; lyp0702@email.swu.edu.cn (Y.L.); zyt20040817@email.swu.edu.cn (Y.Z.); swuzhangye@email.swu.edu.cn (Y.Z.); yyy798620096@email.swu.edu.cn (Y.Y.); wpy20075@email.swu.edu.cn (P.W.); 2 School of Pharmacy, College of Pharmacy, Taipei Medical University, Taipei 110301, Taiwan; lalazsimre@tmu.edu.tw (B.I.); annwong.sverige@gmail.com (A.C.Y.W.); 3 Division of Glycoscience, Department of Chemistry, School of Engineering Sciences in Chemistry, Biotechnology and Health, Royal Institute of Technology (KTH), AlbaNova University Centre, 11421 Stockholm, Sweden

**Keywords:** brown algae, alginate, laminarin, fucoidan, bioactivity

## Abstract

Brown algae (*Phaeophyceae*) have been consumed by humans for hundreds of years. Current studies have shown that brown algae are rich sources of bioactive compounds with excellent nutritional value, and are considered functional foods with health benefits. Polysaccharides are the main constituents of brown algae; their diverse structures allow many unique physical and chemical properties that help to moderate a wide range of biological activities, including immunomodulation, antibacterial, antioxidant, prebiotic, antihypertensive, antidiabetic, antitumor, and anticoagulant activities. In this review, we focus on the major polysaccharide components in brown algae: the alginate, laminarin, and fucoidan. We explore how their structure leads to their health benefits, and their application prospects in functional foods and pharmaceuticals. Finally, we summarize the latest developments in applied research on brown algae polysaccharides.

## 1. Introduction

Algae is an important food source consumed by humans since ancient times. Marine macroalgae, in particular, are important food sources in the coastal regions of East Asia such as China, Korea, Japan, and Indonesia [1]. The global commercial seaweed market was calculated at USD 5.9 billion in 2019 and is anticipated to a compound annual growth rate of 9.1% [2]. Health benefits of seaweed food and snack products are gaining spotlight as vegan sources of protein, lipid and carbohydrates, and demand is expected to boost both for consumption and for further applications. For example, microalgae polysaccharide extracts are used as thickening and gelling agents in the cosmetic and food industries, and the demand is growing particularly in North America and Europe [3]. Among their many uses, the portion directly consumed (excluding thickeners and hydrogels used in food and beverage processing) alone have reached 24 million tons per year, accounting for about 40% of the annual seaweed production [4]. Indeed, the concept of seaweed as healthy food is deeply rooted in many people’s minds. While new applications of polysaccharides derived from marine algae are constantly being discovered, the raising awareness of this ecofriendly, organic, and environmentally sustainable food source further promotes its consumption. Macroalgae are also used in biorefineries; the carbohydrates are converted to high-value byproducts with metabolic engineering approach [5]. The prospects of algae as green, healthy food, and a bioresource is being actively explored.

**Figure 1:**
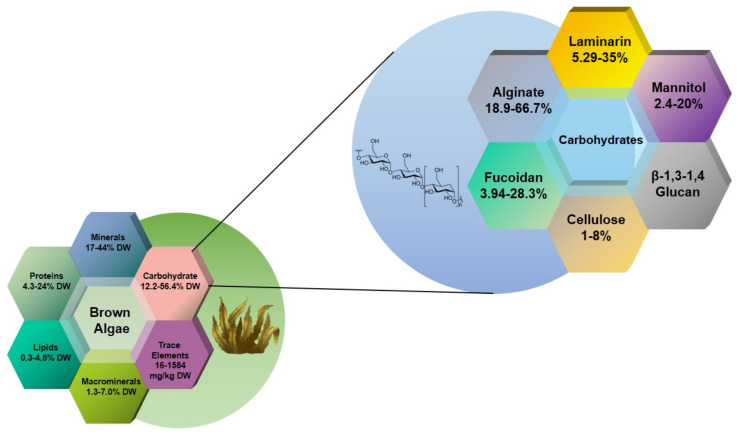
Schematic diagram of the dry matter and carbohydrate composition of brown algae; data summarized from references [9,15,16,17].

Macroalgae are classified into green, red, and brown algae [6]. Brown algae is comprised of 20 classes; the class *Phaeophyceae* alone accounts for over 1800 species and 66% of the total algae consumption [7]. The most common species are the kelps *Laminaria* (kombu), *Undaria* (wakame), and *Macrocystis* [8]. The polysaccharides alginate, laminarin, and fucoidan (Figure 1) account for more than 50% of the total dry weight of brown algae, and can reach up to 70% in some species. Cellulose is the only crystalline component which has been reported in the walls from brown algae so far and it only occurs at 1–8% of algal dry weight [9].Mannitol exists in 2% of laminarin in M-chains, and can also be found on its own, out of the M-chains, in a range of 5–25% of dry weight [10]. It is a sugar alcohol derived from the six carbon sugar D-mannose [11] and appears to be the primary product of photosynthesis [12]. Mixed-linkage-(1,3)-(1,4)-β-D-glucan (MLG) is common in brown algal cell walls. MLG may perform a distinct role in strengthening the cell wall of brown algae [13].The polysaccharides’ proportions and structures vary among species, with some showing markable difference depending on cultivation conditions and harvest seasons [14]. Such heterogeneity may reflect in their diverse biological activities, including anti-inflammatory, antiviral, antioxidant, antitumor, anticoagulant, and hypolipidemic activities, as reported in the literature. This review examines the current knowledge of the biological activity of brown algae polysaccharides and their derivatives as functional foods and bioactive substances. Furthermore, we aim to provide practical strategies and references for developing brown algae-based functional foods and dietary supplements.

## 2. Alginate and Alginate Lyase

**Figure 2:**
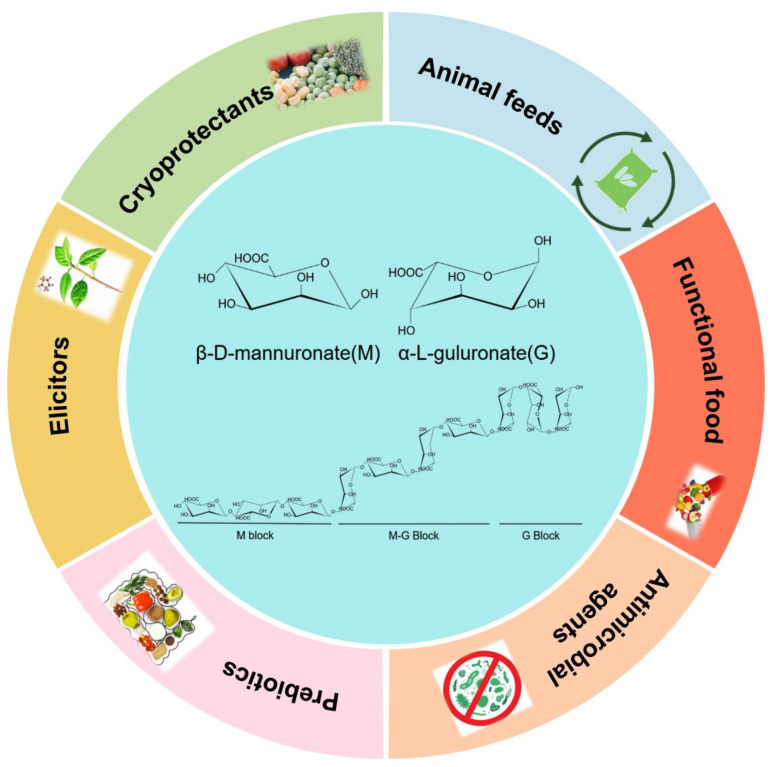
The structure of alginate and the potential applications of alginate oligosaccharides.

Alginate is the predominant polysaccharide component found in the cell walls and intercellular matrix of brown macroalgae. It is a linear polysaccharide composed of two conformational isomer residues: β-d-mannuronic acid (M) and α-l-guluronic acid (G) connected through 1,4-glycosidic linkages [18]. Therefore, the polymer may consist of three types of blocks: homopolymeric sections of consecutive Ms, consecutive Gs, or heteropolymeric sections of randomly arranged M and G units (Figure 2). The ratio of M to G is generally 1:1. Nevertheless, the relative proportions of M and G, as well as their arrangement in the polymer chain, vary according to numerous factors such as the algae species, growth conditions, season, and part of the algae [19]. The M/G ratio of alginate from *Ascophyllum nodosum*, for instance, is about 2:1 [20]. Alginates rich in G residues have higher water solubility than those rich in M residues [21] which also exhibit stronger stiffness and gelling properties due to the presence of metal ions such as Ca^2+^ [22]. 

Alginate oligosaccharides (AOS) are oligomers with a degree of polymerization of 2 to 25, commonly obtained by chemical degradation (such as acid hydrolysis, alkali hydrolysis), physical degradation (such as microwave degradation), enzymatic degradation (alginate lyase), or chemical synthesis. Compared with physicochemical methods, enzymatic degradation of alginate is eco-friendly, energy-saving, selective, and the products are biologically more active [23]. Alginate lyase degrade alginate through β-elimination and produce unsaturated oligosaccharides with double bonds at the non-reducing end [24]. Endolytic alginate lyase have been widely used to produce AOSs with various DPs. For instance, Li et al. found a high activity endo-type alginate lyase from *Pseudomonas* sp. HZJ216 and efficiently produced AOSs with DP of 2–7 [25]. Kim et al. reported an endo-type alginate lyase Alg7D from a marine bacterium *Saccharophagus degradans* 2-40^T^, which produces AOSs DP3–5 [26]. Endo-type alginate lyase Algb from *Vibrio* sp. W13 mainly released oligosaccharides DP of 2–5 [27]. Zhu et al. prepared series of AOSs with DP of 2-5 by using a new alginate lyase Cel32 from *Cellulophaga* sp. NJ-1 [28]. Alginate lyase have the advantage of controlling the predominant DP of AOS products between two and nine without significant amounts of monomers or larger oligomers (DP > 10).

AOSs have been reported to modulate a variety of biological activities and are beneficial to health. Studies have shown that AOSs with different degrees of polymerization have differential biological activity. Therefore, they can be used as antimicrobial, antioxidant, prebiotic, antihypertensive, antidiabetic, antitumor, and anticoagulant agents; their many applications are further discussed below. [29,30,31,32,33,34,35].

### 2.1. Antioxidant Activity

AOSs can scavenge free radicals. The AOS produced by the alginate lyase from *Microbulbifer* (DP: 2–5) was capable to scavenge free radicals (DPPH (2,2-Diphenyl-1-picrylhydrazyl), ABTS+ (2,2′-Azinobis-(3-ethylbenzthiazoline-6-sulphonate), and hydroxyl) and was non-toxic even at high concentrations [36]. Mia et al. found that the AOS prepared by enzymatic method has good antioxidant properties and can completely inhibit the formation of thiobarbituric acid-reactive substances (TBARS) during the iron-induced oxidation of emulsified linoleic acid. In comparison, traditional antioxidant ascorbic acid has only 89% inhibition. For the free radicals ABTS^▪^, ^▪^OH, and O^▪^_2^−^_, polymeric alginate scavenged up to 23, 46, and <1%, while monomeric alginate (represented by glucuronic acid) scavenged up to <1, 25, and 99%, respectively [37]. Due to the conjugated alkenoic acid structure formed during enzymatic depolymerization, AOSs have a higher scavenging ability than similar carbohydrates [37]. The possible mechanism for free radicals scavenging may be a combination of hydrogen abstraction (presumably of hydrogen-bonded hydrogens) and free radical addition to the conjugated olefin acid, resulting in an adduct that is stable through resonance [38].

Compared to chitosan and fucoidan-derived oligosaccharides, AOSs showed higher free radical scavenging capacity [39]. Studies have shown AOSs to play a role in preventing lipid oxidation in skincare emulsions and to scavenge hydroxyl radicals and superoxide anion radicals [40]. In the neuronal PC12 cell model, it was found that AOS pretreatment can block caspase-dependent production of endoplasmic reticulum and mitochondria induced by H_2_O_2_ stress [41]. In mice injured by doxorubicin, AOS pretreatment increases the survival rate through reducing the oxidative stress and inhibiting the expression of gp91phox and 4-hydroxynonenal in the heart [42]. Furthermore, AOSs are also introduced as a new additive in livestock and poultry feed formulation which can effectively improve cellular antioxidative capacity [43]. The free radical scavenging activity of AOSs is tentatively dose-dependent, and that their molecular weight and M/G ratio modulate antioxidative activities. Studies have shown antioxidative activity is negatively correlated with the molecular weight of the oligosaccharide [44,45]. 

### 2.2. Antimicrobial Activity

Hu et al. found that oligo-G and oligo-M (DP: 1–5) obtained by enzymatic hydrolysis had in vitro antibacterial activity against 19 bacterial strains. The antibacterial spectrum of the M oligomer fractions was wider than that of the G oligomers. Within the former, mannuronic acid oligomers with a molecular weight of 4.2 kDa had the highest antibacterial activity and a strong inhibitory effect on *Escherichia coli*, *Salmonella paratyphi*, *Staphylococcus aureus*, and *Bacillus subtilis* [46].

The drug candidate OligoG CF-5/20 is developed by the Norway-based biotech company AlgiPharma. It is a G-enriched alginate oligosaccharide composed of G (85%) and M (15%) blocks. The OligoG CF-5/20 is effective in disrupting and destroying biofilms in a dose-dependent manner. The number of colony-forming units (CFU) in the lungs of infected mice was reduced by 2.5 log; furthermore, 5% OligoG CF-5/20 significantly reduced the minimum biofilm eradication concentration (MBEC) of colistin from 512 to 4 µg/mL after 8 h [47]. OligoG CF-5/20 treatment also reduced *Candida albicans* mycelial infiltration in an in vitro epithelial cell model. OligoG CF-5/20 reduced the expression of *C. albicans* virulence proteins (phospholipase B (PLB2), SAP4 and SAP6) [35], but the mechanism is unclear. Powell et al. also reported AOS exposure to cause changes in biofilm structure, lowering Young’s modulus compared to untreated biofilm. In the untreated control, surface irregularity was higher and resistance to hydrodynamic shear was lower [48]. The results suggested that the observed effect might be caused by OligoG induced changes in the structural characteristics of the extracellular polymers in the bacterial biofilm [48]. Similar effects were found with mucociliary clearance, where lower molecular weight negatively charged G oligomers was found to disrupt the intermolecular interactions of mucus, weakened the viscoelastic properties of mucus, and led to rheological deformation [49].

OligoG CF-5/20 has been proposed as inhalation therapy for the treatment of chronic bacterial respiratory diseases [50]. The oligosaccharides can bind to respiratory mucin, altering its surface charge and the porosity of the three-dimensional mucin network in cystic fibrosis sputum. It has been found that AOSs can act synergistically with the antibiotic azithromycin on wild-type antibiotic-resistant *Pseudomonas aeruginosa*. Azithromycin combined with 2 mg/mL enzymatically produced AOS inhibited the growth of *Pseudomonas aeruginosa*, virulence factors, and biofilm formation controlled by quorum sensing [51]. Pritchard et al. found OligoG CF-5/20 (2%) treatment to induce the destruction of *Pseudomonas aeruginosa* biofilm and colistin to maintain its antibacterial activity. OligoG CF-5/20 did not change the orientation of the alginate carboxyl groups, while mass spectrometry analysis showed the oligomers to reduce pseudomonal quorum-sensing signaling [52]. The gelation of alginate in the presence of divalent cations, such as Ca^2+^, in homopolyguluronic acid, is known to induce changes in coordination of the carboxylate groups [53], which resulting in formation of robust biofilms [54]. However, CD spectra indicated that the orientation of the carboxy groups monitored at ~210 nm were not changed upon mixing OligoG CF-5/20 with high-Mw alginate. This shows that OligoG CF-5/20 combines with Ca^2+^, avoiding the formation of a strong biofilm, so that the colistin can better play an antibacterial effect [52]. Tøndervik et al. found that OligoG (>0.5%) also showed a significant inhibitory effect on mycelial growth in embryonic tube analysis. OligoG (≥2%) alone or in combination with fluconazole significantly hindered fungal biofilm formation. Through the combined treatment, the surface roughness of the cells also increased significantly (*p* < 0.001) [55].

### 2.3. Immunomodulatory and Antitumor Activity

AOSs can enhance immune activity and regulate the function of the immune system in a variety of ways, including regulating the secretion of cytokines and immune-complement molecules. The AOSs produced by depolymerization with alginate lyase increased TNF-α-inducing activity compared to untreated alginate, including the expressions of cytokine-induced TNF-α, granulocyte colony-stimulating factor (CSF), single nuclear cell chemotactic protein-1 (regulated after activating normal T cell expression and secretion), granulocyte macrophages (GM)-CSF, and eosinophil chemokine [56]. AOSs can readily activate macrophages and stimulate TLR4/Akt/NF-κB, TLR4/Akt/mTOR, and MAPK signaling pathways to exert their immune activity [31]. According to the Bio-Plex analysis in RAW264.7 cells, M-rich AOSs tend to have higher immune activity than G-rich oligomers [57]. Uno et al. found that AOSs introduced orally can inhibit the production of IgE and prevent allergic reactions in mice [58]. When administered intraperitoneally, AOSs stimulated the production of 20 cytokines such as granulocyte CSF, monocyte chemoattractant protein-1, IL-6, keratinocyte chemotactic factor, IL-12, and RANTES [59]. AOSs can also induce the production of nitric oxide (NO) by increasing the expression of NO synthase in cells. NO is a multifunctional molecule that can act as a vasodilator, neurotransmitter, inflammatory mediator, and specific immunomodulator [60]. The immunomodulatory activity of AOSs is affected by many factors, e.g., degree of polymerization, purity, M/G ratio, and MG sequence. The unsaturated end-structure achieved by the enzymatic degradation of alginate plays a key role in determining the immunomodulatory activity, as saturated AOSs prepared by acid hydrolysis showed much lower activity. Xu et al. showed that the unsaturated end-structure, molecular size, and M/G ratio of enzymatically produced AOSs affect the activation of macrophages through the NF-κB and MAPK signaling pathways [61,62,63].

Recent studies have also shown AOSs to have antitumor effects. They exert, for instance, an inhibitory effect on the proliferation of human leukemia U-937 cells and produced cytotoxins in human monocytes [56]. AOSs themselves, however, have no direct cytotoxicity to tsFT210 cells. Sulfated AOS derivative with a molecular weight of 3798 Da (sulfation degree of 1.3) has been reported to suppress the growth of solid sarcoma 180 tumor [64]; adding 100 mg/kg AOS, the inhibition rate of solid sarcoma 180 tumor reached 70.4%. It is likely that the AOS and other sulfated derivatives may trigger antitumor effects through organ-mediated immune defense response, especially the immune defense response of the spleen. The AOS of DP 2–10 showed a significant inhibitory effect on the growth of prostate cancer cells. Studies on molecular mechanisms have shown AOSs to attenuate derivatization (α-2,6-sialylation) and reduce ST6Gal-1 promoter activity through the Hippo/YAP/c-Jun signaling pathway [65]. At present, the molecular mechanisms of the contribution of various chemical structural modifications to the antitumor activity of AOSs have not been clarified. Further studies are also needed on the structure-function relationships of antitumor AOSs in targeted cancer therapy.

## 3. Laminarin

**Figure 3:**
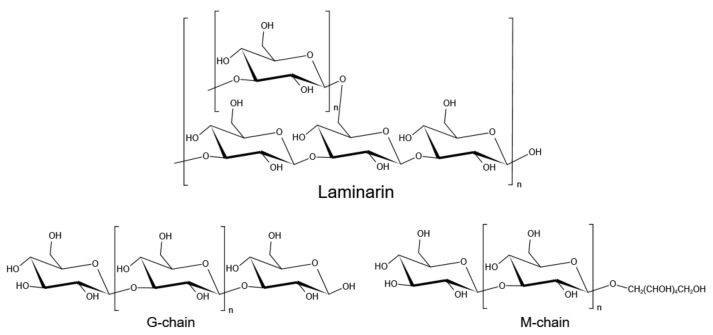
Structures of laminarin.

Laminarin is another major storage carbohydrate of brown macroalgae. It is commonly found in the fronds of *Laminaria* and *Saccharina* macroalgae, although it is also found in *Ascophyllum*, *Fucus* and *Undaria* [7]. Laminarin is a β-glucan, mainly composed of β-1,3-d-glucopyranose residues; the majority of glucose is 6-*O*-branched, while a part of it has β-1,6-intrachain links [66]. Laminarin linked to d-mannitol at the reducing end of the chain is called an M chain, while laminarin without mannitol at the reducing end is a G chain [67] (Figure 3). The ratio of β-1,3- and β-1,6-glycosidic bonds in the polysaccharide depends on the type of algae. For example, laminarin from *Eisenia bicyclis* has a ratio of 2:1 of (1–3) and (1–6) linkage [68]. Laminariales are known to produce high amounts of laminarins, with contents reaching up to 35% of total dry weight, particularly in *L. saccharina* and *L. digitata* [14]. Other reported values of laminarin contents include those of *A. esculenta*, *U. pinnatifida*, *A. nodosum* and *F. serratus* (11.1, 3, 4.5, and up to 19% of total dry weight, respectively) [69,70,71]. The molecular weight of laminarin is about 5 kDa, with a degree of polymerization between 20 and 25 [72,73]. Laminarinases are the enzymes that degrade β-1,3 and β-1,6 glycosidic bonds of laminarin and produce oligosaccharides and glucose, which were classified into endolytic (EC3.2.1.39) and exolytic (EC3.2.1.58) enzymes [74]. The endo-β-1,3-glucanases hydrolyze β-1,3 bonds between adjacent glucose subunits to release oligosaccharides while exo-β-1,3-glucosidase can hydrolyze laminarin by sequentially cleaving glucose residues from the non-reducing end and releasing glucose [75]. For debranching of laminarin, β-1,6-glucanases randomly hydrolyze β-1,6 glycosidic bonds and release gentio-oligosaccharides or glucose [76]. Endo laminarinases were widely applied to produce oligosaccharides. Recently, Kumar et al. reported a thermostable laminarinase belongs to GH81 from *C. thermocellum* which can hydrolyze laminarin into a series of oligosaccharides (DP2 to DP7) [77]. Badur et al. reported four laminarinases from *Vibrio breoganii* 1C10, of which *Vb*GH16C can hydrolyze laminarin to oligosaccharides of DP8 and DP9, and *Vb*GH17A can hydrolyze laminarin into a series of laminarin oligosaccharides (DP4 to DP9) [78]. Wang et al. characterized a bifunctional enzyme from GH5 subfamily 47 (GH5_47) in *Saccharophagus degradans* 2-40^T^ and identified as a novel β-1,3-endoglucanase (EC 3.2.1.39) and bacterial β-1,6-glucanase (EC 3.2.1.75). This bifunctional laminarinase can degrade both the backbone and branch chain of laminarin, and is also active on hydrolyzing pustulan which is a linear β-1,6-glucan. This enzyme also showed transglycosylase activity toward β-1,3-oligosaccharides when laminarioligosaccharides were used as the substrates [79]. The above findings provide more possibilities for the green preparation of biologically active oligosaccharides.

Laminarins and laminarin oligosaccharides are recognized for their various biological activities; they have shown to stimulate innate immunity [80], stimulate antitumor responses [81,82], increase resistance to infections [83], promote wound repair [84], and enhance the immune response of macrophages [85]. Laminarins can be used to activate macrophages, leading to immune stimulation, antitumor, and wound-healing activities [86]. Furthermore, they can be partially or fully fermented by endogenous gut microbiota [87]. Consequently, they have good prospects for application in the field of functional foods and biomedicine.

### 3.1. Antioxidant and Antimicrobial Activities

Studies have shown of the crude extracts of laminarin from *L. hyperborea* and *A. nodosum* to remove DPPH free radicals effectively, with clearance rates of 87.6 and 93.2%, respectively. Compared to extracts obtained with water solvents, acid-extracted laminarin was showed to have higher antioxidant activity [88].

Laminarin-rich seaweed extracts are found to have inhibitory effects against both Gram-positive (such as *Staphylococcus aureus* and *Listeria monocytogenes*) and Gram-negative (*E. coli* and *Salmonella typhimurium*) bacterial strains. Notably, the inhibitory rate of *A. nodosum* extract against *Salmonella typhimurium* can reach 100%. Laminarin-rich extracts obtained using ultrasound and acid solvents had minimum inhibitory concentrations (MIC) of 13.1 mg/mL for *E. coli* and *S. typhimurium* and 6.6 and 3.3 mg/mL for *S. aureus* and *L. monocytogenes*, respectively [88]. Therefore, the polysaccharide can be applied in the preparation of antibacterial products such as edible packaging materials and even wound dressings.

### 3.2. Antitumor and Anticoagulant Activity

Several studies have demonstrated the significant antitumor and anticancer activities of laminarin and laminarin oligosaccharides [89]. The underlying mechanisms include apoptosis and the inhibition of cancer cell colony formation [90]. Different concentrations of laminarin have been used to treat human colon cancer LoVo cells and the intracellular reactive oxygen species (ROS), pH, intracellular calcium ion concentration, mitochondrial permeability transition pore, mitochondrial membrane potential, and Cyt-C, Caspase-9 and Caspase-3 expression levels were analyzed. The studies have found kelp polysaccharides to induce the apoptosis of human colon cancer LoVo cells through the mitochondrial pathway [91,92]. The polysaccharide did not show direct cytotoxicity, but exhibited significant antitumor activity on SK-MEL-28 human melanoma cells and it could effectively inhibit the colony formation of HT-29 cells [93,94].

Laminarin oligosaccharides can inhibit the proliferation of human tissue lymphoma cell line (U937 cells) by stimulating monocytes to produce cytokines [95]. Specific enzyme products with high content of 1,6-linked glucopyranose residues (laminarin oligosaccharides with DP 9–23) have shown significant anticancer activity and can inhibit the colony formation of melanoma and colon cancer cells [96,97]. Sulfated laminarins (LAMS) with a sulfate content of 45.92% proved to inhibit the growth of LoVo cells more significantly than laminarin, suggesting the better antitumor activity of LAMS. Accordingly, enzymatic hydrolysis and molecular modification provide new ideas for the production of laminarin derivatives with high antitumor activity [98].

The anticoagulant activity of *Laminaria* sp. extract was first reported in 1941 [99]. Although laminarin is a non-sulfated polysaccharide in seaweed, its sulfated products showed anticoagulant activity [100]. Many studies have been published on the extraction and modification of laminarin sulfate from algae in the genus *Laminaria*. If each glucose residue has an average of two sulfate groups, the anticoagulant activity of the preparation reaches 25–30% of that of standard heparin [101], while the activity of sulfonic acid derivatives appears to be higher than that of sulfate esters [102]. A derivative of laminarin with 1.83 sulfate groups per glucose unit showed 33% of the potency of heparin in rabbits, although it was extremely toxic to guinea pigs [103]. This suggests that laminarin sulphate might be effective in the prevention and treatment of cerebrovascular diseases.

### 3.3. Anti-Inflammatory and Immunostimulatory Activity

Studies have shown that β-glucans cause reduced recruitment of inflammatory cells and decreased secretion of inflammatory mediators in liver tissues through direct effects on immune cells or indirect effects as dietary fibers [104]. Laminarin significantly increases the release of inflammatory mediators, such as hydrogen peroxide, calcium, nitric oxide, monocyte chemoattractant protein-1, vascular endothelial growth factor, leukemia-inhibitory factor, and granulocyte colony-stimulating factor, and enhances the expression of signal transducer and transcriptional activators [86]. Recent studies have found laminarin to induce positive effect of decreasing mitochondrial activities without cytotoxicity caused by oxidative stress by regulating the interaction between glycans and receptors on the skin cell surface [105].

### 3.4. Prebiotic Activity

The prebiotic properties of algae polysaccharides enable them to play an important role in regulating human intestinal health [106]. For laminarin, it has been confirmed in vitro that it cannot be hydrolyzed by hydrochloric acid under physiological conditions, nor by homogenates of the human digestive system [14,107]. Since laminarin is resistant to hydrolytic enzymes in the human upper digestive tract, it can reach the intestinal flora [108]. Animal experiments have shown that adding laminarin to the diet of mice can significantly reduce the Firmicutes to Bacteroidetes ratio in the intestines, indicating that it can enhance the high-energy metabolism of the intestinal microbiota to reduce the side-effects of high-fat diets [109]. In addition, laminarin oligosaccharides are beneficial for the growth of *Bifidobacterium animalis* and *Lactobacillus casei*, also increasing the production of short-chain fatty acids, such as lactic acid and acetic acid [110].

## 4. Fucoidan

**Figure 4:**
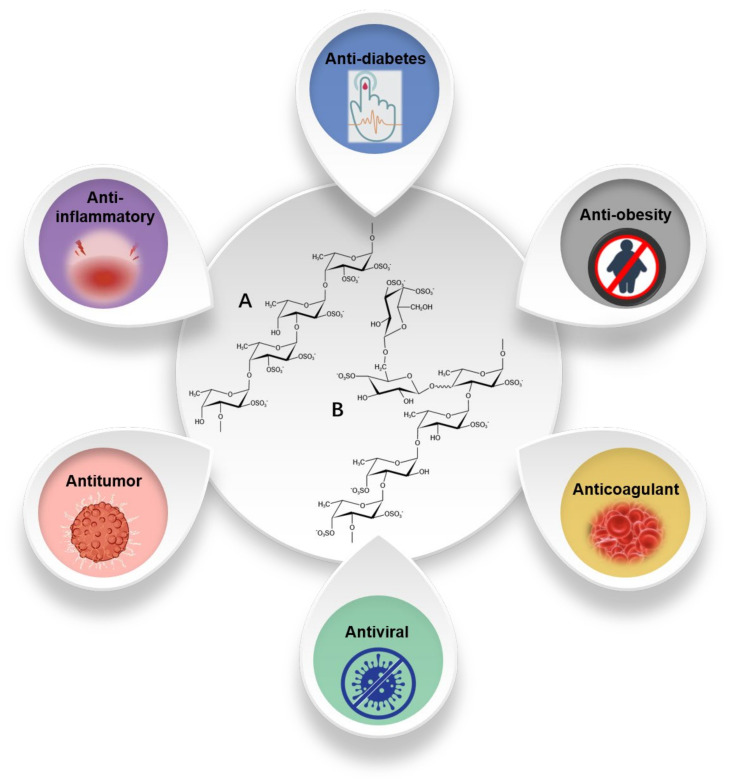
Structure and biological effects of fucoidan (A: *Ascophyllum nodosum* and *Fucus vesiculosus*; B: *Saccharina japonica*, adapted from literature [119,120,121]).

Fucoidan is a sulfated polysaccharide that consists mainly of fucose repeating units besides several other monosaccharide residues. It is commonly found in brown seaweed [111,112], and has also been reported in echinoderms and some lower plants [113]. Fucoidan typically acts as a structural polysaccharide in the cell walls of brown macroalgae, with its relative amount ranging between 4 and 8% of the total dry weight [114]. Since fucoidan was first isolated in 1913, the structure of fucoidans from different brown seaweeds has been studied. Seaweed fucoidan is a heterogeneous material, with varying composition of carbohydrate units and non-carbohydrate substituents [115]. Fucoidan is mainly composed of fucose and sulfate groups (Figure 4). For example, the fucoidan from bladder wrack (*Fucus vesiculosus*) has a simple composition and contains only fucose and sulfate groups (44.1% fucose, 26.3% sulfate) [116]. In addition, it might also contain other monosaccharides (mannose, galactose, glucose, xylose, etc.), uronic acid, and even acetyl groups and proteins. For example, the fucoidan from *Fucus vesiculosus* contains 84% fucose, 6% xylose, 7.3% galactose, and 2% mannose [117]. The fucoidan found in *Fucus distichus* is composed of 51.6% fucose, 2.7% xylose, 1.5% galactose, 0.7% mannose, and 0.2% glucose [118]. Comprehensive analysis concluded that the fucose content of fucoidans is in the range of 4.45–84%, besides 1.44–49% galactose, 0.2–45% glucose, and 0.3 to 16% xylose and mannose. As a heterogeneous polymer, fucoidan exhibits considerable structural diversity that makes it difficult to draw general conclusions. Moreover, its structure cannot be described solely based on monosaccharide composition.

The structural variety of fucoidans is to a large extent related to the different types of brown algae they are found in. Generally, α (1→3) and/or (1→4) glycosidic bonds constitute the main chain of the macromolecules, dominating in most backbone structures. The presence of sulfate groups at the C-2, C-4 and or C-3 position is another important feature [94,122,123,124,125,126,127]. Due to the structural heterogeneity of fucoidans, the degradation of fucoidan requires a large set of enzymes of different activities and specificities [128]. Fucoidanase are mainly from marine bacteria, invertebrates and sometimes fungi. Similar to the above mentioned polysaccharide-degrading enzymes, endo-type fucoidanase produce fuco-oligosaccharides while exo-type fucosidase leads to the formation of mono- or oligosaccharides with a small degree of polymerization [129]. Natalie et al. purified a new fucoidanase and hydrolyzed fucoidan without desulfation to form oligosaccharides ranging from 10 to 2 fucose units plus fucose [130]. Dong et al. discovered a new α-L-fucosidase from marine bacterium *Wenyingzhuangia fucanilytica*, and found that Alf1_Wf was capable of hydrolyzing α-1,4-fucosidic linkage and synthetic substrate. Besides, Alf1_Wf could act on partially degraded fucoidan [131]. Compared to other brown polysaccharides, there are few studies on the enzymatic degradation of fucoidan and the function of fuco-oligosaccharides, whereas the functional investigation of biological activities, such as anti-obesity, antivirus, antitumor, antidiabetic, and antioxidative effects has been widely proven. It is generally believed that fucoidan can become an important substance in the functional food and nutrition and health industries [132,133].

### 4.1. Antitumor Activity

Fucoidan has significant antitumor activity against liver cancer, stomach cancer, cervical cancer, lung cancer, and breast cancer [113,134,135,136,137,138]. The underlying mechanism includes the inhibition of tumor cell proliferation, stimulating tumor cell apoptosis, blocking tumor cell metastasis, and enhancing various immune responses [136,139,140,141]. Low molecular weight fucoidan (LMWF), for instance, triggers G1-block and apoptosis in human colon cancer cells (HCT116 cells) through ap53-independent mechanisms [142]. Through the assessment of microtubule-associated proteins and the accumulation of Beclin-1, fucoidan is also found to induce autophagy in human gastric cancer cells (AGS cells) [143]. The polysaccharide induces the apoptosis of HTLV-1-infected T-cell lines mediated by cytostatics that downregulate apoptosis protein-2. The use of fucoidan in vivo thus severely inhibits the tumor growth of subcutaneously transplanted HTHT-1-infected T-cell lines in immunodeficient mice [138]. In addition, fucoidan activates the caspase-independent apoptotic pathway in MCF-7 cancer cells by activating ROS-mediated MAP kinase and regulating the mitochondrial pathway mediated by Bcl-2 family proteins [144]. Similarly, fucoidan has shown antitumor activity against PC-3 (prostate cancer), HeLa (cervical cancer), A549 (alveolar cancer), and HepG2 (hepatocellular carcinoma) cells [145].

### 4.2. Antiviral and Anti-Inflammatory Activity

Fucoidans isolated from different seaweed species have potential antiviral activity. For instance, they can inhibit the replication of enveloped viruses, including the human immunodeficiency virus (HIV) and the herpes simplex virus (HSV) [146]. According to Queiroz et al. [147], fucoidans from *Dictyota mertensii*, *Lobophora variegata*, *Spatoglossum schroederi*, and *Fucus vesiculosus* inhibit the HIV reverse transcriptase (RT) enzyme, while other studies have shown that they also reduce the amount of the HIV-1 p24 antigen [148]. Compared with other antiviral drugs currently used in clinical medicine, the inhibitory effect of fucoidan is accompanied by lower cytotoxicity. According to one potential mechanism, fucoidan prevents viruses from entering the cells by changing the characteristics of the cell surface. The polysaccharide may also directly interact with viral enzymes or viral proteins on the surface of the pathogen.

Many studies have reported the blocking effect of fucoidan on HSV infection. Fledman et al. isolated different fucoidan components from *Leathesia difformis* and verified the selective antiviral activity of different components against HSV-1 and HSV-2 [149]. Fucoidan extracted from *Undaria pinnatifida* has shown antiviral activity against 32 HSV clinical strains, including 12 ACV-resistant (4 HSV-1 and 8 HSV-2) and 20 ACV-susceptible ones. Judging by the survival rate and lesion score, oral fucoidan can protect mice from HSV-1 infection by stimulating cytotoxic T lymphocytes, natural killer activity, and neutralizing antibodies [150].

The above findings clearly indicate the potential antiviral activity of seaweed fucoidans, which can also strengthen the immune response of the host and achieve multi-channel and multi-level regulation of the immune system [151,152]. The polysaccharide is able to prevent virus transmission by directly inhibiting virus replication and stimulating innate and adaptive immune defense functions. The immunomodulatory activity of fucoidan is another hot research topic. Numerous studies have already confirmed fucoidan to exhibit an anti-inflammatory effect through immune regulation (Table 1). This involves the polysaccharide binding to different receptors, e.g., the Toll-like receptors (TLRs) of monocytes, such as dendritic cells (DCs) and macrophages, and thereby initiating the release of pro-inflammatory factors: cytokines and chemokines. They also suppress the expression of NO synthase (iNOS) and cyclooxygenase (COX)-2 at the protein level, and dose-dependently inhibit the production of nitric oxide (NO) and prostaglandin E2 (PGE2).

Fucoidan can enhance the various beneficial effects of lactic acid bacteria on immune function by improving Th1/Th2 immune balance [162], and can also treat gastric mucosal damage caused by oral aspirin through its ability to regulate immune response and reduce ulcers’ inflammation [163]. During in vivo experiments, Li et al. evaluated the potential inhibitory activity of fucoidan on the myocardial ischemia-reperfusion (I/R) model in rats. The results showed a significant effect by modulating the inflammatory response through the inactivation of high mobility group box 1 (HMGB1) and nuclear factor kappa B (NF-κB) [164].

It has been reported that the destruction of connective tissue during inflammatory diseases such as chronic wounds, chronic inflammation, or rheumatoid arthritis is a result of a continuous supply of inflammatory cells and increased production of inflammatory cytokines and matrix proteases [165]. Selectins expressed on endothelial cells, leukocytes, and platelets contribute to the interaction of leukocytes and platelets on the side of vascular injury, thereby enhancing the inflammatory response during the arterial response to injury [166]. Fucoidan can effectively inhibit the interaction between selectins and their ligands leading to reduced inflammation at an early stage. Therefore, fucoidan use seems beneficial for treating certain inflammations accompanied by uncontrolled extracellular matrix degradation. The above studies have laid the preclinical foundation for the development of fucoidans as a new generation of polysaccharide immunomodulators.

### 4.3. Antidiabetic Activity

Studies have shown that fucoidan can also exhibit antidiabetic effects by reducing postprandial hyperglycemia and pancreatic β-cell damage, increasing insulin secretion, and regulating glucose metabolism to reduce blood sugar [167,168]. Fucoidan has a significant inhibitory effect on the three starch-hydrolyzing enzymes; it is a non-competitive inhibitor of α-amylase and amyloglucosidase, while being a competitive inhibitor of α-glucosidase [169]. Its inhibitory mechanism lies in the formation of hydrogen bonds [170]: the hydroxyl groups of fucoidan, especially the ones at the C-terminus that may be connected to fucose, can easily form hydrogen bonds with the amino acids of the two enzymes. The negatively charged oxygens of the sulfated groups of the polysaccharide (and the ones connected to C-2 and/or C-3 in particular) further facilitate the formation of hydrogen bonds or salt bridges with the proteins, resulting in strong interactions, thereby inhibiting the enzyme. Furthermore, inhibition of dipeptidyl peptidase-IV (DPP-IV) is one of the possible mechanisms involved in the antihyperglycemic activity of fucoidan. Dipeptidyl peptidase-IV (DPP-IV) is an enzyme that is involved in the inhibition of the rapid degradation of incretin hormones, which prevents postprandial hyperglycemia. Inhibiting DPP-IV prolongs the action of incretins, which reduces glucose production and increases insulin production [171]. Fucoidan can be used as a dipeptidyl peptidase-IV (DPP-IV) inhibitor to block DPP-IV action thereby prolonging the half-life and biological activity of incretin hormones [172], which play a crucial role in glucose homeostasis by promoting α and β cell function [173]. It also downregulates the gastric emptying and gastric acid secretion to reduce the postprandial glucose level [174,175]. Olga N. Pozharitskaya et al. have found a concentration-dependent inhibition of the enzyme DPP-IV by fucoidan at the concentration range of 0.02–200 µg/mL, The IC50 was 11.1 µg/mL and the maximum inhibition degree was 60–75% [176].

In addition, fucoidan may have a positive effect on antidiabetics by reducing β cell damage in the pancreas and increasing insulin secretion. According to a complex mechanism, the polysaccharide enhances the activity of sirtuin 1, thereby inducing deacetylation and upregulation of FOXA2 and p-FOXO-1 to promote the expression of PDX-1 and its regulation of insulin synthesis, thereby reducing β cell apoptosis and dysfunction in mice [177]. Furthermore, fucoidan is able to prevent the occurrence of diabetic nephropathy (DN) associated with spontaneous diabetes by inhibiting the NF-κB signaling pathway and lowering blood sugar in a non-toxic way [178]. It has also been found that a combination of fucoidan and traditional Chinese medicine has a beneficial effect on hyperglycemia and DN in rats [179].

### 4.4. Other Biological Activities

Heparin is a highly sulfated polysaccharide found in mammalian tissues and has been used as an anticoagulant for more than 50 years [180]. However, the clinical use of heparin is known to cause various side effects, such as excessive bleeding, thrombocytopenia, mild transaminase elevation, and hyperkalemia [181]. Therefore, it is necessary to find alternative drugs with safe and effective anticoagulant properties. It is worth noting that fucoidan has shown effectiveness for blood clotting, and many studies suggest it as an alternative to heparin [182,183]. Through studying the anticoagulant activity of fucoidans isolated from nine species of brown seaweed, the ones from *Ecklonia kurome* and *Hijikia fusiforme* were found to have the strongest effect in terms of thromboplastin time (TT) and activated partial thromboplastin time (aPTT) [184]. The mechanism of fucoidan action differs from that of heparin since it can be used in the cases where the application of heparin itself, for some reason or other, is ineffective. The anticoagulant action of fucoidans (as well as that of heparin) can be quickly blocked by the intravenous introduction of biocompatible cationic polymers such as protamine sulfate and VIM-DEMC (a synthetic copolymer of 1-vinylimidazole with diethyl maleate) [185]. Fucoidans may inhibit thrombin activity by directly acting on the enzyme or through the activation of thrombin inhibitors, including antithrombin III and heparin cofactor II [186].The position of the sulfate group on the sugar residues was found to be an important factor, with the concentration of C-2 sulfated and C-2,3 disulfated residues considerably affecting anticoagulant activity [123].

Fucoidan also has a positive effect in treating and preventing obesity. It has been shown to suppress the formation of 3T3-L1 adipocytes, thus inhibiting fat accumulation, by downregulating fatty acid binding proteins, acetyl-CoA carboxylase, and peroxisome proliferation-activated receptor γ. [187]. Furthermore, *Fucus vesiculosus*-derived fucoidan was found to hinder fat accumulation in 3T3-L1 adipocytes by stimulating lipolysis through increased hormone-sensitive lipase expression and reduced glucose uptake [188].

At present, there is limited information available regarding the antiallergic effect of fucoidan. Recent studies have shown that the orally administered polysaccharide suppresses allergic symptoms by promoting the expression of galectin-9 mRNA and serum galectin-9 levels, thereby preventing immunoglobulin E (IgE) binding to mast cells [189].

## 5. Conclusions

This review summarized the physicochemical and structural properties of polysaccharides and oligosaccharides derived from brown algae. Their structure and composition determine their biological activity and thereby their nutritional and therapeutic potentials. Although more is now known regarding their biological activities in vitro and significant advance has been made in their extraction from natural sources and modifications, further structural-activity investigation is necessary. Sustainable technologies must be established for the purification of the polysaccharides and the production of oligosaccharides, minimizing energy and chemical consumption while allowing upscaling of consistent quality and freedom from side-effect causing impurities. Lastly, research on catalytic enzymes, including alginate lyase, laminarinase, fucoidanase, and fucosidase, with high stability and desired substrate specificity is needed to enable the production of high-purity oligosaccharides with uniform structure and degrees of polymerization. Progress in enzyme and metabolic engineering will further promote the utilization of brown algae polysaccharides in the food and pharmaceutical industries.

## Figures and Tables

**Table 1:** Monosaccharide composition, molecular weight, concentration, sulfation degree and anti-inflammatory mechanism of different fucoidans.

Brown Seaweed	Monosaccharide Composition	Molecular Weight	Concentration	Sulphate Content (%)	Mechanisms	References
*Cladosiphon novae-caledoniae*	Fucose 73 mol%, Xylose 12 mol% Mannose 7 mol%	-	19.35 ng/µL; 80.64 ng/µL	14.5%	Inhibited COX-1 and COX-2	[153]
*Sargassum horneri*	Polyphenols 3.9%	Mw > 30 kDa	25–100 µg/mL	12%	Decreased production of TNF-α, IL-6, NO and PGE2	[154]
*Laminaria japonica*	Fucose 79.49% Xylose 1.08% Mannose 1.84% Galactose 16.76% Rhamnose 0.82%	104.3 kDa	25 µg/mL	30.72%	Decreased production of TNF-α, IL-1β, IL-6, NO, iNOS, and COX-2 expression; downregulation of MAPK and NF-κB signaling pathways	[155]
*Turbinaria decurrens*	Fucose 59.3% Xylose 11.4% Galactose 12.6% Mannose 9.6%	-	50 mg/kg	23.51%	Reduced the expression of genes of COX-2, IL-1β, the NF-κB signaling pathway	[156]
*Turbinaria ornata*	Fucose 86.4 mol% Rhamnose 0.4 mol% Galactose 1.7 mol% Glucose 0.8 mol%	-	25–100 µg/mL	38.3%	Suppressed the expression of COX-2 and pro-inflammatory cytokines in LPS-induced RAW 264.7 macrophages	[157]
*Undaria pinnatifida*	Fucose 50.9% Xylose 4.2% Galactose 44.6% Mannose 0.3%	-	50 mg/kg;150 mg/kg		Reduced the COX-2 expression dose dependently	[112]
*E* *c* *klonia cava*	Fucose 77.9 mol% Rhamnose 2.3 mol% Galactose 10.1 mol% Glucose 2.2 mol% Xylose 7.5 mol%	-	50–100 µg/mL	39.1%	Reduced NO production and levels of TNF-α, IL-1β, and IL-6	[158]
*Fucus vesiculosus*	Molar rate 1:0.03:0.02:0.04:0.2:1.2 for Fucose, Galactose, Mannose, Xylose, Uronic acid, and sulfate	-	30–60 mg/kg	27%	Inhibition of COX, hyaluronidase, and MAPK p38 enzymes.	[159]
*Cladosiphon* *okamuranus*	Fucose 30.9% Xylose 0.7% Glucose 2.2% Uronic acid 23.4%	-	4.0 mg/kg	15.1%	Inhibition of neutrophil extravasation into peritoneal cavity	[115]
*Fucus vesiculosus*	Fucoidan	-	0–100 mg/mL		Inhibited the release of nitric oxide, IL-1b, TNF-a, prostaglandin E2 and monocyte chemoattractant protein-1 by inhibiting NF-κB, Akt and MAPK kinases activation	[160]
*Sargassum hemiphyllum*	Fucose 210.99 mmol/g	-	100 mg/mL	38.99.4%	Inhibition of IL-1b, TNF-a, and reduction of IL-10, IFN-c in production LPS treated cells	[161]
*Macrocystis pyrifera*	Fucose 25.77% Galactose 3.93% Glucose 1.14% Mannose 1.12% Xylose 0.84% Uronic acid 5.54%	-	5-100 μg/mL	27.32%	Delayed the apoptosis and promote pro-inflammatory cytokine production in human neutrophils	[151]
*Ascophyllum nodosum*	Fucose 39.8% Galactose 3.37% Glucose 0.88% Mannose 0.72% Xylose 3.68% Uronic acid 1.72%	-	50–100 μg/mL	24.07%	Delayed the apoptosis and promote pro-inflammatory cytokine production in human neutrophils	[151]
